# Biostabilization and Transport of Cohesive Sediment Deposits in the Three Gorges Reservoir

**DOI:** 10.1371/journal.pone.0142673

**Published:** 2015-11-11

**Authors:** Hongwei Fang, Mehdi Fazeli, Wei Cheng, Lei Huang, Hongying Hu

**Affiliations:** 1 Department of Hydraulic Engineering, State Key Laboratory of Hydro-science and Engineering, Tsinghua University, Beijing, China; 2 Department of Civil Engineering, Yasouj University, Yasouj, Iran; 3 School of Environment, Tsinghua University, Beijing, China; Peking UIniversity, CHINA

## Abstract

Cohesive sediment deposits in the Three Gorges Reservoir, China, were used to investigate physical and geochemical properties, biofilm mass, and erosion and deposition characteristics. Biofilm cultivation was performed in a recirculating flume for three different periods (5, 10 and 15 days) under ambient temperature and with sufficient nutrients supply. Three groups of size-fractionated sediment were sequentially used, including 0–0.02 mm, 0.02–0.05 mm and 0.05–0.10 mm. Desired conditions for erosion and deposition were designed by managing high bed shear stress at the narrow part of upstream flume and low shear stress at the wide part of downstream flume. Biostabilization and transport characteristics of the biofilm coated sediment (bio-sediment) were strongly influenced by the cultivation period, and the results were compared with clean sediment. The bio-sediment was more resistant to erosion, and the mean shear stress was increased by factors of 2.65, 2.73 and 5.01 for sediment with 5, 10 and 15 days of biofilm growth compared with clean sediment, resulting in less sediment being eroded from the bed. Simultaneously, the settling velocity was smaller for bio-sediment due to higher organic content and porosity (i.e., lower density). Additionally, there was a smaller probability of deposition for sediment with a longer cultivation period after erosion, resulting in more retention time in aquatic systems. These results will benefit water management in natural rivers.

## Introduction

In aquatic systems many contaminants of concern are associated with and transported by cohesive sediment [1]. Biostabilization and transport of cohesive sediment are critical to the aquatic environment and bed morphology. Sediments in a trophic water column provide excellent substrata for microorganism colonization [2–4]. The secretion of metabolic products (e.g., extracellular polymeric substances, EPS) causes the formation and growth of biofilm on the sediment surfaces [5, 6]. Biofilm is a complex matrix of living microorganisms and their metabolic products. Here, sediment particles coated with biofilm are defined as bio-sediment to distinguish them from clean sediment.

The development of biofilm can influence the sediment properties (e.g., size gradation, morphology, density and stability) and their transport processes [7, 8], as well as affect sediment associated contaminant transport [9]. Field investigations have shown that biofilm contributes a major part of the cohesiveness of sediments [10] and influences the sediment dynamics through binding fine-grained sediment, changing water content, and increasing organic content, etc. [11, 12]. Thus, biofilm growth could significantly enhance biostabilization, with biofilm-infused bed sediments requiring more energy for erosion relative to clean sediment. Studies on biogenic sediment stabilization were first carried out in marine systems [11, 13–16], followed by some recent studies in freshwater systems (i.e., streams and lakes) [12, 17–19]. However, the biofilm effects on biostabilization and transport of cohesive sediment in reservoirs have rarely been reported.

In recent decades, a great number of reservoirs have been built in China, significantly affecting the sediment fluxes and causing profound and irreversible changes to river system functions [20, 21]. Most reservoirs, such as the Three Gorges Reservoir (TGR), employ the strategy of storing clear water and discharging turbid water to control sedimentation and maintain capacity [22]. In non-flood seasons, the water level is kept at the normal pool level. Thus, the flow slows down, resulting in a significant accumulation of both sediment and nutrients at the bed surface [23], which increases the biofilm formation and growth rate. Biofilm can permeate void spaces and promote inter-particle linkages, and the resultant increased level of attachment within and between deposited flocs can lead to increased bed stability [12]. When the reservoir discharges turbid water in flood seasons, the bio-sediment is eroded into the overlying water, causing many serious aquatic environment problems in the reservoir [24]. Therefore, knowledge of the biostabilization and transport of bio-sediment is essential for the operation of the TGR.

In this study, flume experiments were designed to enhance our understanding of the biostabilization and transport of bio-sediment in reservoirs. Biofilm cultivation was performed in the flume for different periods, and the biofilm formation and growth were then characterized. Subsequently, the bio-sediment was eroded under certain hydrodynamic conditions. The biofilm effects on the biostabilization and transport of the bio-sediment were effectively investigated through addressing the erosion process (e.g., incipient velocity and bed shear stress), suspended sediment concentration (SSC) distribution, and the deposition process (e.g., variation of size gradation and settling behavior), etc. These results will provide valuable references for the operation of the TGR.

## Materials and Methods

### Ethics statement

No specific permissions were required for sampling activities in these locations. We confirmed that these field studies did not involve endangered or protected species.

### Study area and sample collection

The Three Gorges Reservoir, which is located in the Yangtze River, started operation in 2003, and more than 1.0×10^8^ t of sediment is deposited in the reservoir annually [20, 25]. Simultaneously, significant amounts of bio-available nutrients and organic matter are trapped in the reservoir and accumulate at the bed surface. The dramatic alterations of the sediment and nutrient characteristics adversely impact the water environment of the TGR, and serious eutrophication has occurred, especially in the tributaries (e.g., Xiangxi River) [26]. The increased accumulations of bio-available nutrients and organic matter have significantly increased the presence and abundance of biofilms in the TGR. Here, cohesive sediment and river water were collected from the TGR. Surface deposits of fine sediment were collected with a plastic scoop, immediately refrigerated at 4°C and then transported within one week to the laboratory of Tsinghua University, Beijing, where they were studied using a recirculating flume.

### Experiment design

#### Flume setup

**Fig 1:**
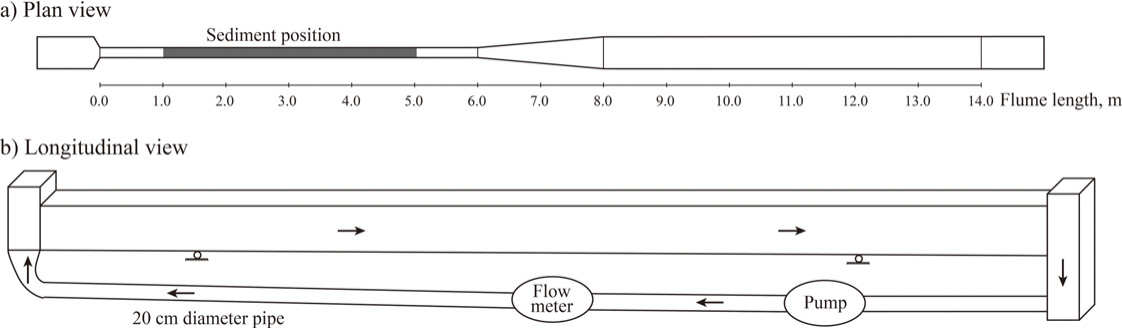
(a) Top view and (b) longitudinal view of the recirculating flume, including an upstream channel (0 m < *x* < 6 m), an expansion region (6 m < *x* < 8 m) and a downstream channel (8 m < *x* < 14 m). The sediment is shown by the gray bar from 1 m to 5 m.

A flume with an overall length of 14 m and a width of 0.5 m was modified to provide the desired conditions for both erosion and deposition [23], as shown in Fig 1. A constant width of 0.16 m was maintained in the upstream channel between *x* = 0 m and *x* = 6 m (where *x* is the distance from the flume inlet). In the region 6 m < *x* < 8 m, the channel width was extended from 0.16 m to 0.5 m. Downstream of the expansion region, the channel had a constant width of 0.5 m. Moreover, the flume had a slope of 0.0025, and the experiment water was recirculated within the flume by a pump system [23]. The experiment was designed to observe sediment erosion in the upstream channel and deposition in the downstream channel. In particular, a flow damper was arranged in the front of the flume to trap the recirculated suspended load, and a regulation gate was deployed at the end of the flume for flow condition control (Fig 1).

#### Biofilm cultivation

Sediment samples were placed in the upstream channel of the flume (i.e., the region from 1 m to 5 m), as shown by the gray bar in Fig 1A. It is evident that fine sediment is conducive to biofilm attachment, whereas coarse sediment contains less biofilm. Here, three groups of size-fractionated sediment, including 0–0.02 mm, 0.02–0.05 mm and 0.05–0.10 mm, were sequentially used to consider the effects of sediment size on biostabilization. It is important to keep in mind that sediment with a size of 0–0.10 mm is the main component of deposits in the TGR. Then, two ends of the region from 1 m to 5 m were closed and an appropriate amount of river water was added to a depth of approximately 6 cm for biofilm cultivation.

To promote biofilm growth, nutrients were added into the river water to ensure a relatively high trophic level. The nutrient solution compositions were proposed by Wu et al. [27] and optimized by Zhao [28] as: glucose (C_6_H_12_O_6_)—0.5 g/L, potassium dihydrogen phosphate (KH_2_PO_4_)—0.05 g/L, sodium bicarbonate (NaHCO_3_)—1.0 g/L, anhydrous magnesium sulfate (MgSO_4_)—0.05 g/L, ammonium chloride (NH_4_Cl)—0.1 g/L, and calcium chloride (CaCl_2_)—0.015 g/L. Biofilm growth was performed for three growth periods (5, 10 and 15 days) under ambient temperature, with an average value of approximately 19.6°C. The experiment water was refreshed everyday using a siphon tube to replenish the nutrients.

#### Erosion experiment

A bio-sediment bed was formed in the upstream channel of the flume. After biofilm growth of 5, 10 or 15 days, the whole flume was slowly filled with experiment water to a depth of 10 cm to avoid disturbing the bio-sediment bed. Then, the pump system started working, and the discharge was gradually increased by the control system to observe the incipient motion of bio-sediment. A new bio-sediment bed was formed from the erosion of bio-sediment in the upstream channel of the flume and the deposition in the downstream channel. As described above, the erosion experiments involved three groups of size-fractionated sediment, and three different cultivation periods, that is, totally nine experiment scenarios were carried out. Simultaneously, the erosion experiment of clean sediment (i.e., biofilm growth of 0 day) was also performed for comparison.

During the experiment, water samples were taken for sediment concentration analysis using a specifically designed instrument [23]. The time interval between each sampling was approximately 15 min. Note that the first sampling was implemented right after detecting suspended sediment, i.e., just a few minutes after the incipient motion of bio-sediment. For each sampling, water samples were collected every 2 m along the flume. The sediment concentration was measured using a photoelectric sediment concentration meter (CYS-III, Nanjing Hydraulic Research Institute, China). Moreover, the bed morphology was measured using the MASATOYO electronic profile indicator (EPI, Japan), with an interval of 10 cm along the axis of the flume and eight discrete points in each section. Morphology was measured twice for each experiment, i.e., initial bed surface (at the beginning of the erosion experiment) and final bed surface (a few hours after terminating the experiment), as an indicator of overall erosion conditions. Moreover, measurements of the hydrodynamic parameters, including discharge, water level and flow velocity, were implemented during the experiment.

### Sediment samples analyses

#### Physical and geochemical properties

Cohesive sediment samples were collected from TGR and submitted for physical and geochemical analysis. The grain size distribution was determined using a laser scattering particle size distribution analyzer (LA-920, HORIBA). The mineral composition was evaluated via the X-ray powder diffraction method using a Philips X’pert PW3040-PRD diffractometer with a Cu X-ray source operated at 40 kV and 50 mA. The concentrations of major elements (including N, O, Na, Mg, Al, Si, P, S, Cl, K, Ca, Fe and Cu) were determined via X-ray fluorescence spectrometry, and certified reference materials USGS GXR-1, GXR-2, GXR-4 and GXR-6 were analyzed at the beginning and the end of each batch of samples.

#### Microscopy observation

Environmental scanning electron microscopy (ESEM), which can be utilized in the wet or partially hydrated state, is a new type of instrument with high precision for the observation of surface microstructure and ultrastructure and has been widely applied in the fields of environment, microbiology and materials science [29]. Generally, there are three working modes for ESEM, i.e., environmental, high vacuum, and low vacuum modes. Specifically, the environmental mode can accept a wet sample, which is essential for the morphology observation of environmental samples. In this study, the FEI Quanta 200 FEG ESEM (FEI Company, Eindhoven, the Netherlands) was used for the morphology observation of bio-sediment, which was collected from the upstream channel of the flume after a certain period of biofilm growth. Then, bio-sediment that was highly hydrated with poor electrical conductivity could be observed directly without damaging the architecture, and the real images of morphology could be obtained.

#### Biofilm mass

Biofilm is an amorphous complex matrix comprising living microorganisms (e.g., bacteria, microalgae and fungi) and their metabolic products (e.g., EPS). The sediment loss on ignition (LOI) is a traditional and convenient measure of organic content [30, 31] that can be regarded as an indirect estimation of biofilm mass. Here, bio-sediment samples gathered from the flume were first dried at 105°C, and then the LOIs were determined using a Muffle furnace at 550°C for 8 h. The amount of biofilm mass was expressed as the mass of organic matter per gram of sediment (mg/g).

## Results and Discussion

### Biofilm formation and analysis

#### Physical and geochemical properties

The geochemical properties and mineral composition of the initial sediment will influence biofilm formation by affecting sediment properties. The mineral composition of the cohesive sediment deposits in the TGR are shown in Table 1. It was found that quartz accounted for the largest percentage (50%), followed by albite (22%), while only a small percentage of the clay minerals (i.e., 5% muscovite and 5% clinochlore) was observed. Thus, sediment is an assemblage of minerals, potentially resulting in complicated morphology and surface charge distribution [32, 33], which are beneficial for microorganism attachment and biofilm formation. Moreover, the major element composition of the sediment sample is presented in Table 2, including the mass percentage and atomicity percentage. It was observed that O, Si and N were the major elements composing the sediment sample, totally accounting for more than 95.5% of the sediment. Moreover, approximately 2.5% of Al and Fe (i.e., corresponding to the Fe/Al (hydr)oxides), which were critical to the nutrients and contaminants adsorption, were observed.

**Table 1:** Mineral composition of cohesive sediment deposits in the TGR (%).

Mineral	Formula	Percentage
Muscovite	KAl_2_Si_3_AlO_10_(OH)_2_	5.00±4.32
Amphibole	Al_3.2_Ca_3.4_Fe_4.02_K_0.6_Mg_6_NaSi_12.8_O_44_(OH)_4_	3.00±0.71
Quartz	SiO_2_	50.20±14.06
Microcline	K(AlSi_3_)O_8_	4.40±1.85
Albite	(Na,Ca)Al(Si,Al)_3_O_8_	22.20±18.71
Calcite	CaCO_3_	8.25±1.09
Dolomite	CaMg(CO_3_)_2_	7.40±4.03
Clinochlore	(Mg,Al,Fe)_6_(Si,Al)_4_O_10_(OH)_8_	4.50±2.69

**Table 2:** Major element composition of cohesive sediment deposits in the TGR (%).

Element	Mass percentage	Atomicity percentage	Element	Mass percentage	Atomicity percentage
N	5.53±0.70	7.77±0.97	S	0.17±0.24	0.10±0.14
O	51.18±4.19	62.89±3.62	Cl	0.13±0.10	0.07±0.06
Na	0.30±0.21	0.26±0.19	K	0.65±0.27	0.33±0.14
Mg	0.71±0.19	0.58±0.17	Ca	0.64±0.10	0.31±0.05
Al	2.46±0.92	1.80±0.69	Fe	2.27±0.83	0.80±0.31
Si	35.38±2.98	24.89±2.66	Cu	0.58±0.42	0.18±0.13
P	0.01±0.00	0.01±0.00			

#### Biofilm formation

**Fig 2:**
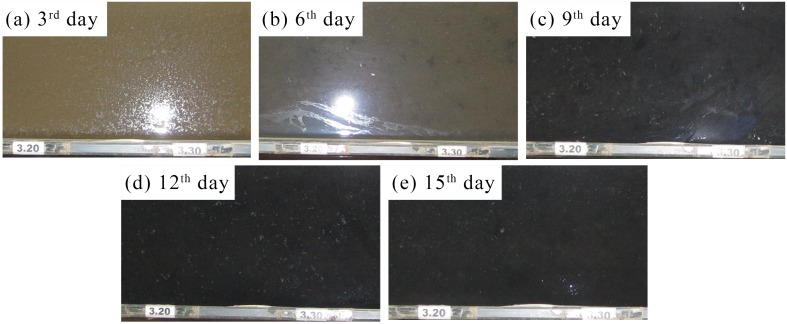
Typical photographs of the biofilm growth with the corresponding cultivation period. The number in each image represents the cultivation period, i.e., images (a)-(e) correspond to cultivation periods of 3, 6, 9, 12 and 15 days, respectively.

Photographs of the biofilm growth with the cultivation period are shown in Fig 2, where images (a)-(e) correspond to cultivation periods of 3, 6, 9, 12 and 15 days, respectively. The images in Fig 2 clearly show that cultivation period obviously affects the quality and quantity of biofilm growth. As shown in Fig 2A, no biofilm formation was observed before the 5th day, and then biofilm growth occurred gradually. The biofilm growth started with points of apparent dark color (Fig 2B) and overspread to the entire cultivation region after the 9th day (Fig 2C). Hereafter, the apparent dark color of the biofilm had few changes (Fig 2C–2E), while changes in the odor and thickness of the biofilm continued until the last day. As with the biofilm formation, a thin layer of foam-like materials developed on the water surface.

**Fig 3:**
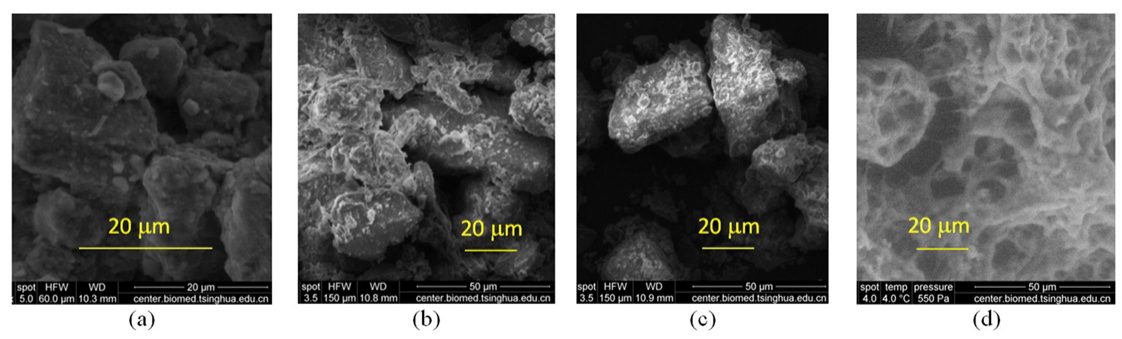
Environmental scanning electron microscopy photos of sediment samples after 15 days of biofilm growth. (a) Environmental mode, (b) high vacuum mode using the dried sample after environmental mode, (c) high vacuum mode using the clean sediment, (d) foam, which developed on the water surface during cultivation.

After 15 days of biofilm growth, a mature biofilm was formed on the bed surface. ESEM was then used to observe the morphology of the bio-sediment, as shown in Fig 3. First, the bio-sediment was observed using the environmental mode with a relative humidity of 100%. The real ultrastructure of samples could be directly observed without damaging the architecture. Thereafter, each sample was tested using the high vacuum mode together with the clean sediment. These ESEM images showed more organic coatings and inter-particle linkages for the bio-sediment relative to the clean sediment; thus, the biofilm-infused bed sediment required more energy to undergo erosion. It seemed that the foam-like materials (Fig 3D) facilitated biofilm formation via partial deposition on the bed surface.

#### Biofilm mass

**Fig 4:**
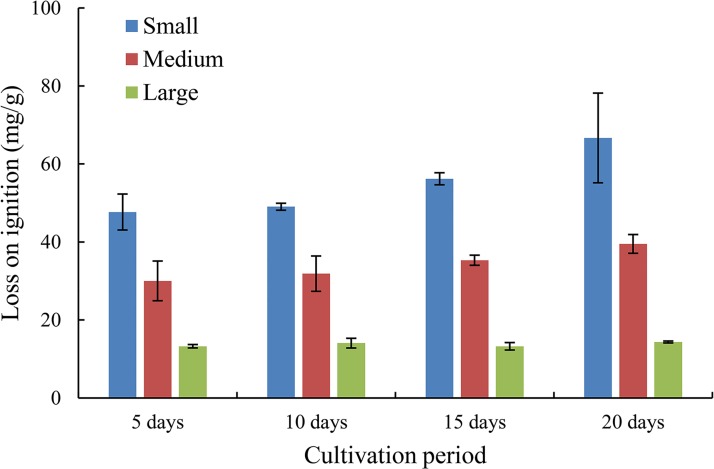
Relations between loss on ignition (LOI) and cultivation period for three groups of size-fractionated sediment: small (0–0.02 mm), medium (0.02–0.05 mm) and large (0.05–0.10 mm).

A simple estimate of the biofilm growth on the bed surface, expressed as mass of organic matter per gram of sediment (LOI), is shown in Fig 4, including three size-fractionated sediments, i.e., small (0–0.02 mm), medium (0.02–0.05 mm), and large (0.05–0.10 mm). Fine sediment had an overall higher level of organic content relative to coarse sediment (i.e., the average values were 54.90, 34.18 and 13.74 mg/g for the small, medium and large sizes, respectively), which was consistent with the intuitive feeling. For each group of size-fractionated sediments, the values of LOI increased with increasing cultivation period, especially after 10 days of biofilm growth, implying the significant effects of cultivation period on biostabilization. Considering the increasing rate, one may deduce that the effect of cultivation period on LOI is more intense for small sediments, while relatively weaker effects may exist for medium and large sediments. After 20 days of biofilm growth, the LOI values reached 66.67, 39.50 and 14.36 mg/g for the small, medium and large sizes, respectively. In view of the intensive biofilm growth, i.e., a more significant impact on biostabilization, the following analyses were mostly focused on the small sediments group (0–0.02 mm).

### Biostabilization and transport of bio-sediment

#### Biofilm erosion process

**Fig 5:**
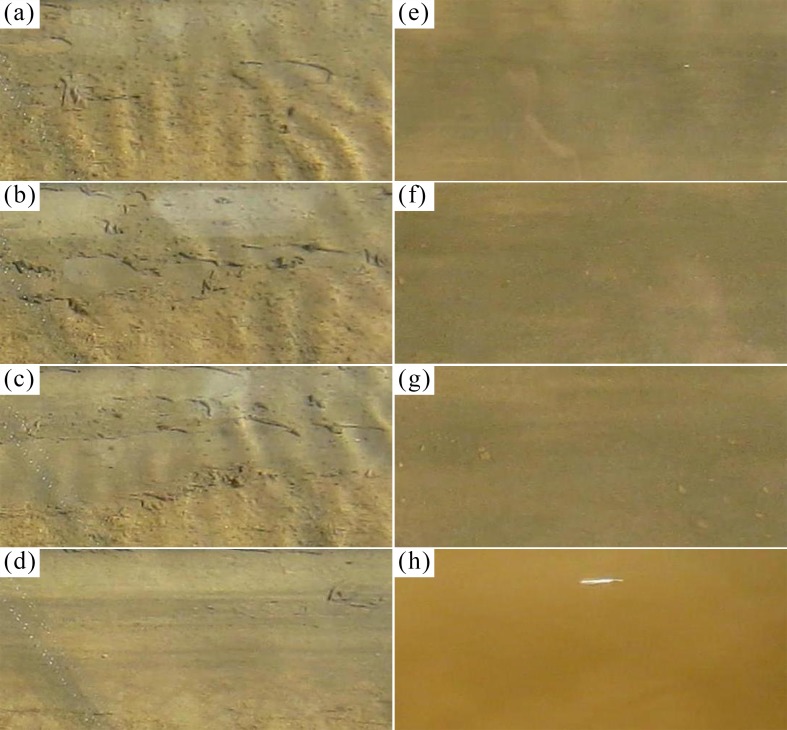
Sequential images displaying the erosion process of bio-sediment, i.e., from the destruction of the biofilm mat to the suspension of lower layers. The entire time interval (a-h) is nearly one hour. The images belong to three time groups: a-d, e-g and h. The time interval between adjacent images of each group is 20 s, and it is 30 min between different groups.

For bio-sediment, an additional adhesive force acts on the sediment particles through biofilm growth, resulting in a different erosion behavior relative to clean sediment. Fig 5 shows some sequential images of biofilm detachment and the subsequent erosion of the underlying sediment, with a cultivation period of 5 days. Note that these images belong to three groups, i.e., Fig 5A–5D, 5E–5G and 5H, with a time interval of 20 s between adjacent images in each group, while 30 min between different groups. The whole period lasted approximately 1 h. Biofilm acted as a mat on the bed surface and began to erode before the underlying sediment. As shown in Fig 5A–5D, biofilm erosion started with the occurrence of small fractures, followed by rolling up upon itself and then detaching completely. Thereafter, flocculated particles were eroded with increasing bed shear stress, and their saltation and sliding motions motivated the underlying sediment to suspend. Fig 5E–5G shows some bio-sediment sliding on the bed, and a large amount of sediment was suspended in Fig 5H. The change in color from dark to yellowish brown in these photos was probably a sign of clean sediment erosion, implying that the biofilm only developed in the surface layer.

Several studies have demonstrated that biostabilization can significantly increase the energy required to erode sediment [12, 13], i.e., a higher bed shear stress to reach a threshold of bio-sediment motion. Thus, a quantitative method to determine the incipient velocity of bio-sediment is needed. In our previous work [34], a predictive formula for the incipient mean velocity of bio-sediment was established, simultaneously considering the cohesive force between sediment particles and the adhesive force generated by biofilm, i.e.,

$$\frac{U_{c}^{2}}{gD}=\frac{\gamma_{s}-\gamma }{\gamma }\left[6.25+41.6\frac{H}{H_{a}}\right]+\left[111+740\frac{H}{H_{a}}\right]\frac{H_{a}\delta }{D^{2}}+\frac{A\left(t\right)}{\gamma D}$$ (1)

where *U*_*c*_ is the incipient mean velocity in the section (cm/s); *g* is the acceleration of gravity (cm/s^2^); *D* is the particle diameter (cm); *γ* and *γ*_*s*_ are the bulk density of water and sediment, respectively (g/s^2^/cm^2^); *H* is the water depth (cm); *H*_*a*_ is the head of water, equivalent to the atmospheric pressure (cm); *δ* is the diameter of a water molecule (= 3×10^−8^ cm). The parameter *A*(*t*), which represents the biofilm effects, is a function of the cultivation period *t* (in weeks) and particle diameter *D*, expressed as

$$A\left(t\right)=18.6D^{-0.503}\cdot t\cdot e^{1-0.355t}$$ (2)

Thus, both the properties of biofilm (i.e., cultivation period *t*) and sediment particles (i.e., particle diameter *D*) were considered in Eq (1).

**Fig 6:**
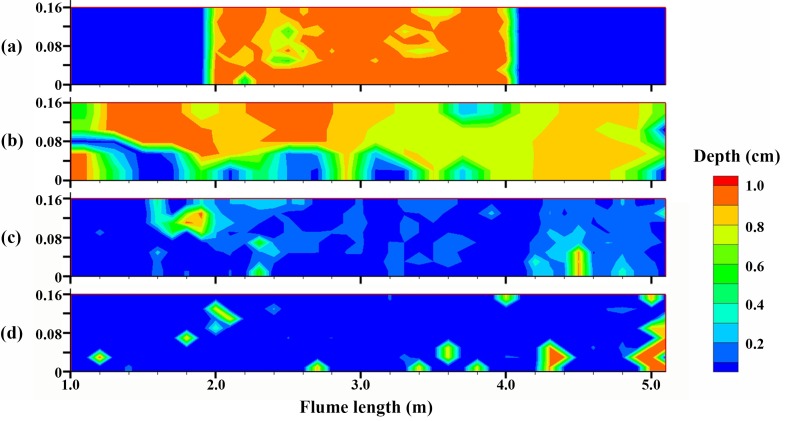
Erosion maps of bio-sediment based on the morphology measurement in the region from 1 m to 5 m. Different colors represent different erosion depths; (a) clean sediment, (b) 5 days, (c) 10 days and (d) 15 days of biofilm growth.

The incipient velocities *U*_*c*_ for bio-sediment with a cultivation period of 5, 10 and 15 days were calculated at *x* = 2.4 m and compared with the clean sediment, as shown in Table 3. Moreover, the observed values were also presented, corresponding to the first sampling that was implemented right after detecting suspended sediment. Thus, it was estimated that the observed *U*_*c*_ was slightly larger than the calculated value. Overall, the calculated incipient velocities were comparable to the observed values. Moreover, greater incipient velocities were observed for the bio-sediment relative to the clean sediment, implying an increase of resistance to erosion due to biofilm growth. The incipient velocity increased with increasing cultivation period, i.e., the bio-sediment with the cultivation period of 15 days had the largest *U*_*c*_, followed by 10 days of biofilm growth and then 5 days. Thus, less erosion was expected for bio-sediment with a longer cultivation period. Fig 6 shows the erosion maps based on the measured morphology, representing the overall erosion conditions. It was evident that less bio-sediment was eroded after the flume ran for a longer cultivation period. Statistics showed that approximately 73.8%, 68.3%, 28.2% and 14.8% of the total sediment was eroded for experiments using sediment with cultivation periods of 0, 5, 10 and 15 days, respectively.

**Table 3:** Comparison between the calculated and observed incipient velocities at *x* = 2.4 m for different experiments.

Group	Cultivation period	Discharge (m^3^/h)	Observed incipient velocity (m/s)	Calculated incipient velocity (m/s)
1	Clean sediment	19.1	0.58	0.54
2	5 days	22.8	0.65	0.60
3	10 days	31.1	0.69	0.64
4	15 days	58.0	0.77	0.66

#### Bio-sediment transport

**Fig 7:**
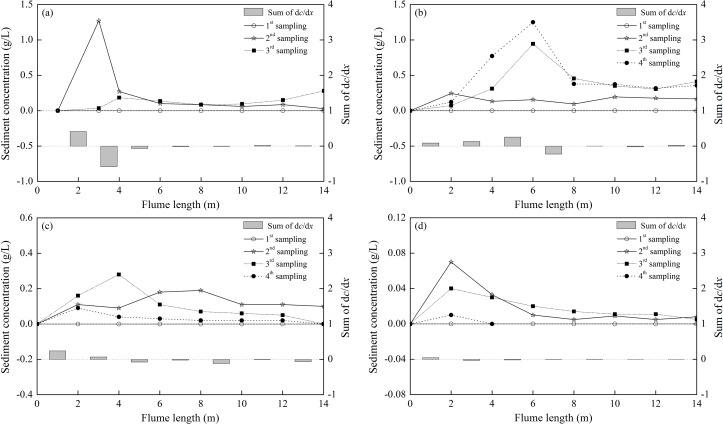
The temporal and spatial variations of sediment concentration for different cultivation periods. (a) Clean sediment, (b) 5 days, (c) 10 days and (d) 15 days of biofilm growth. In each image, the sediment concentrations are modified according to the value at *x* = 0 m, and the sum of d*c*/d*x* represents the net sediment erosion (positive) or deposition (negative) between two adjacent sections.

The temporal and spatial variations of sediment concentration (denoted as *c*) for different cultivation periods (0, 5, 10 and 15 days) are shown in Fig 7. The sediment concentrations were modified according to the value at *x* = 0 m. In each image, the curves correspond to four different samplings, expressed by hollow circles and stars and solid squares and circles, respectively. Overall, a shared trend was observed for different cultivation periods, i.e., a peak sediment concentration was observed in the upstream channel of the flume where erosion occurred, and then it decreased along the flume due to deposition. Considering the effects of biofilm growth on the maximum SSC, Fig 7 revealed that clean sediment had a maximum value of approximately 1.3 g/L, followed by 5, 10 and 15 days of biofilm growth with values of 1.25 g/L, 0.3 g/L and 0.07 g/L, i.e., a decrease of 1.5%, 78% and 94.5% compared to clean sediment, respectively. More adhesive force due to biofilm growth represented greater biostabilization, simultaneously resulting in smaller suspended sediment concentrations. To quantify the ascending or descending manner of a specified curve, its slope could be calculated. The summation of all such slopes at each length interval represented the net sediment erosion (i.e., sum of d*c*/d*x*>0) or deposition (i.e., sum of d*c*/d*x*<0). Similarly, it was found that the maximum positive value of the sum of d*c*/d*x* was related to clean sediment, followed by 5, 10 and 15 days of biofilm growth. Because the sum of d*c*/d*x* identified the intensity of erosion, greater positive values represented more sediment erosion.

The bed shear stress associated with skin friction, which is responsible for the entrainment of sediment into suspension, could be expressed as *τ* = *γR*_*b*,*s*_ *S*_*f*_, where *R*_*b*,*s*_ is the hydraulic radius of the bed region associated with skin friction and *S*_*f*_ represents the energy slope. *R*_*b*,*s*_ was calculated using the method described by Viparelli et al. [35]. Here, *τ*_*Er*_ and *τ*_*De*_ represent the bed shear stresses in the erosion and deposition regions, i.e., *x* = 4.0 m and 12.0 m of the flume length, respectively. Moreover, Δ*c*_*Er*_ = *c*_*max*_-*c*_*inlet*_ (i.e., a positive value) denotes the change of sediment concentration in the erosion region, while Δ*c*_*De*_ = *c*_*outlet*_-*c*_*max*_ (i.e., a negative value) represents the change in the deposition region. Subscripts *inlet*, *outlet* and *max* represent the beginning, the end and the location of maximum concentration along the flume length, respectively.

**Fig 8:**
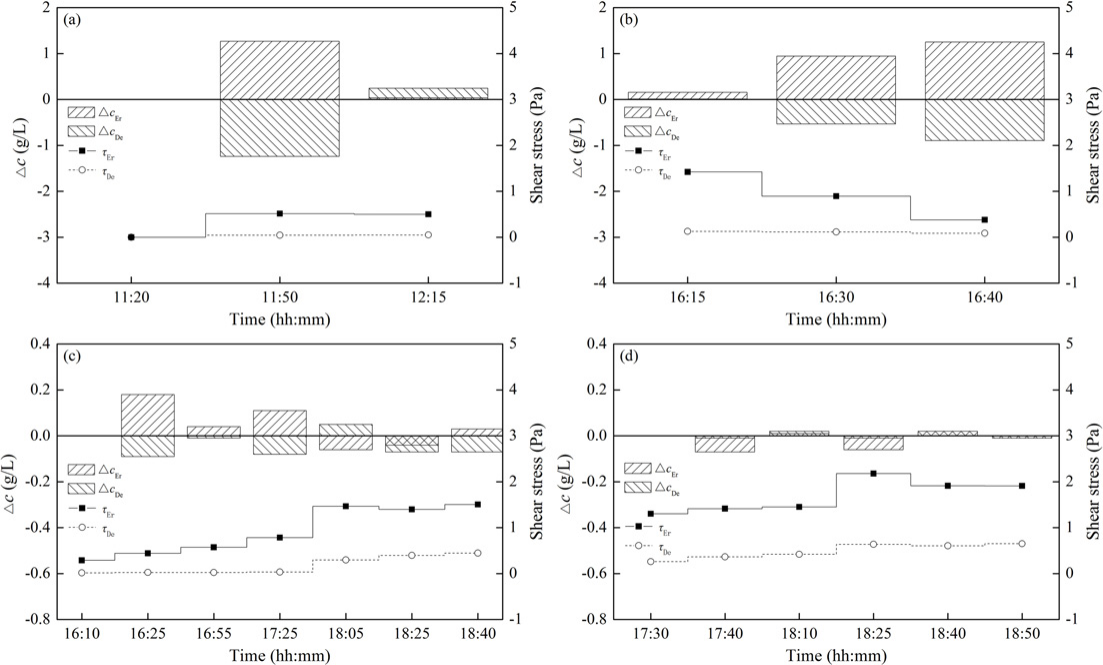
Calculated shear stress and the change of sediment concentration for different cultivation periods. (a) Clean sediment, (b) 5 days, (c) 10 days and (d) 15 days of biofilm growth. Δ*c*_*Er*_ and Δ*c*_*De*_ specify the concentration changes in the erosion and deposition regions, respectively.

Fig 8 shows the calculated bed shear stress and the change of sediment concentration for different cultivation periods. The *X*-axis represents the time during the erosion experiment. A larger *τ*_*Er*_ was observed for the sediment with a longer cultivation period. For example, a shear stress of approximately 2 Pa was needed for sediment with 10 and 15 days of biofilm growth (Fig 8C and 8D), while less than 1 Pa was needed for clean sediment and 5 days of biofilm growth (Fig 8A and 8B). Statistics showed that there was an increase of a factor of 2.65, 2.73 and 5.01 of the mean shear stress for sediment with 5, 10 and 15 days of biofilm growth compared with clean sediment. As stated in the literatures, shear stresses of 0–0.4 Pa and 0.4–1.0 Pa were derived for 14 days of biofilm growth by Stone et al. [1] and Watanabe et al. [36], respectively, while here, a shear stress of 1.3–2.2 Pa was calculated for 15 days of biofilm growth (Fig 8D). These discrepancies were probably caused by the specific cultivation conditions in this study, e.g., the addition of nutrients in the experiment water, and the day by day replenishment of nutrients, leading to a deeper and stronger biofilm growth. It was verified that parts of the biofilm still remained unchanged after the flume ran.

Meanwhile, it was apparent that more time was needed for the erosion of sediment with a longer cultivation period. As shown in Fig 8, the total erosion time for clean sediment and 5 days of biofilm growth was less than 1 h, while approximately 2 h was needed for sediment with 10 and 15 days of biofilm growth. Similarly, the absolute values of Δ*c*_*Er*_ and Δ*c*_*De*_ were much larger for clean sediment and 5 days of biofilm growth, i.e., more than 1 g/L at some specific time, while less than 0.2 g/L was observed for sediment with 10 and 15 days of biofilm growth. All of these results illustrated that the stronger biofilm growth due to a longer cultivation period enhanced the biostabilization, which significantly increased the energy required for sediment erosion and resulted in a greater resistance to erosion.

#### Variation of size gradation and settling analysis

**Fig 9:**
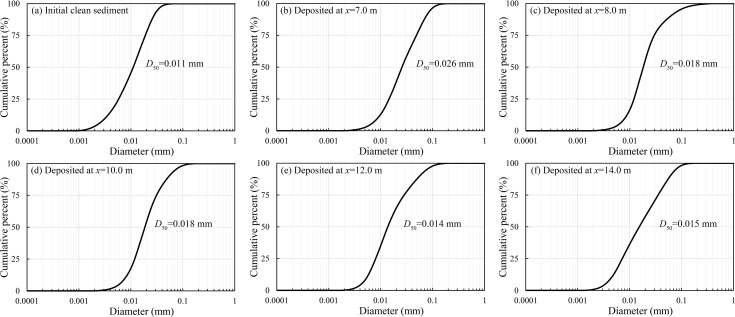
Size gradation curves of the initial clean sediment (a) and the deposited sediment at *x* = 7.0, 8.0, 10.0, 12.0 and 14.0 m (b-f) for the erosion experiment of 15 days of biofilm growth.

The grain size distribution of deposited sediment (i.e., the eroded flocs) was compared to the initial clean sediment in Fig 9, indicating the effects of biofilm growth on sediment size. Here, the results for a cultivation period of 15 days were presented. A clean sediment sample was taken from the initial bed (before biofilm growth) at *x* = 2.4 m, while deposited sediment samples were gathered from successive positions at *x* = 7.0, 8.0, 10.0, 12.0 and 14.0 m after the flume ran. Comparing the characteristic particle sizes, it was evident that deposited sediment had a larger size relative to clean sediment, i.e., at least 65% and 150% increase of the median size and maximum size, respectively. However, these measured sediment sizes were still smaller than the expected values for bio-sediment predicted by Shang et al. [8], which could be partly due to the destruction of flocculated structure before deposition. Although the sediment size increased significantly during the biofilm growth, it decreased gradually due to the flow turbulence after being eroded from the bed surface. Furthermore, the median sizes of 0.026, 0.018, 0.018, 0.014 and 0.015 mm were observed for deposited sediment samples at *x* = 7.0, 8.0, 10.0, 12.0 and 14.0 m, respectively, i.e., larger bio-sediment deposited first, followed by smaller ones.

Approximately 42.4%, 33.6%, 10.2% and 2.2% of the total sediment deposited in the flume for experiments using sediment with cultivation periods of 0, 5, 10 and 15 days, respectively, while approximately 73.8%, 68.3%, 28.2% and 14.8% of the total sediment was eroded, as described in the section of *Biofilm erosion process*. We defined a settling rate as the ratio between the amounts of deposition and erosion. Then, the derived settling rates of clean sediment and bio-sediment cultivated for 5, 10 and 15 days were 57.4%, 49.2%, 36.2% and 14.9%, respectively, implying a smaller probability of deposition for sediment with a longer cultivation period once eroded, which was likely related to the characteristics of eroded flocs. Generally, bio-sediment that eroded into the water column formed flocs with high organic content and porosity, as well as low density, resulting in small settling velocities. Thus, bio-sediment with a longer cultivation period could remain in the water column for a longer period of time despite its greater resistance to erosion, i.e., a challenge for water management. These results were consistent with the conclusions reported by Stone et al. [1] and Shang et al. [8].

### Implications for water management

In aquatic systems, many contaminants of concern are associated with and transported by cohesive sediment [1]. The present study showed that biofilm growth on the sediment surface could enhance the biostabilization, significantly increasing the energy required for sediment erosion, and could affect sediment-associated contaminant transport. In flood seasons, the water level of the TGR is kept at a normal pool level of 175 m with a water depth of approximately 100 m, resulting in a significant accumulation of both sediment and nutrients at the bed surface [23] and providing favorable conditions for biofilm formation and growth. Kuang et al. [37] studied the phytoplankton in the TGR during the period before and after impoundment and reported that the species richness and cell densities of algae were considerably increased after the operation of the TGR. In flood seasons, the bio-sediment will be eroded into the overlying water due to the increase of flow velocity. Flume experiments showed that bio-sediment with a longer cultivation period had a smaller probability of deposition after erosion due to the higher organic content and porosity (i.e., lower density). Therefore, bio-sediment will remain in the water column for a long time once the incipient velocity *U*_*c*_ is exceeded, i.e., a potential challenge for water management of the TGR.

### Conclusions

1. Biofilm mass increased with increasing cultivation period, and a greater increasing rate was observed for fine sediment relative to coarse sediment. ESEM images showed more organic coatings and inter-particle linkages for the bio-sediment relative to clean sediment, which could significantly increase the energy required to erode sediment.

2. Biofilm growth significantly enhanced the biostabilization, i.e., a longer cultivation period resulted in a greater resistance to erosion. Larger shear stresses were observed for the erosion of sediment with longer biofilm growth periods, i.e., a 2.65-, 2.73- and 5.01-fold increase of the mean shear stress for sediment with 5, 10 and 15 days of biofilm growth compared to clean sediment. Correspondingly, the clean sediment had the maximum value of the SSC (i.e., the most sediment erosion), followed by sediment that was cultivated for 5, 10 and 15 days.

3. Biofilm growth caused the aggregation of sediment particles, forming flocs with high organic content and porosity, as well as low density. Thus, bio-sediment with a longer cultivation period had a lower settling velocity, resulting in more retention time in aquatic systems after erosion.
